# Waste to energy through sustainable bioenergy and biohydrogen production from lignocellulosic waste using microbial fuel cell and microbial electrolysis cell

**DOI:** 10.1038/s41598-026-50075-3

**Published:** 2026-05-19

**Authors:** Mahmoud Bayoumi, Mohamed S. Hassouna, Ahmed A. Hussien, Hanan Moustafa Abdallah Moustafa

**Affiliations:** 1 Department of Biotechnology, Institute of Graduate Studies and Research, Alexandria University, Alexandria, Egypt; 2 Department of Environmental Studies, Institute of Graduate Studies and Research, Alexandria University, Alexandria, Egypt

**Keywords:** Biotechnology, Energy science and technology, Engineering, Environmental sciences

## Abstract

The accumulation of agricultural lignocellulosic waste, specifically rice straw, and the environmental impact of fossil fuel combustion necessitate the development of sustainable waste-to-energy technologies. Bioelectrochemical systems enable a simultaneous recovery of energy and the treatment of organic waste utilizing a mixed culture of bacteria. This study investigates for the first time the performance of rice straw-derived substrates with different levels of complexity, i.e. rice straw, rice straw hydrolysate, and xylan, to simultaneously produce bioelectricity and biohydrogen, with the objective of clarifying the influence of substrate characteristics on system efficiency. Microbial fuel cells (MFC) result indicated that xylan exhibited the highest performance, achieving a maximum cell potential of 852 ± 27 mV and a power density of 8.88 ± 0.27 W/m², representing a 2.4-fold increase over the control. This was supported by a high Chemical Oxygen Demand (COD) removal efficiency of 97.88 ± 1.32%. The higher values of power density are attributed to the employment of a mixed bacterial culture (sludge) that has already adapted to the degradation of lignocellulosic biomass, hence boosting its utilization and conversion to electrons, resulting in higher electricity generation. Electrochemical analysis indicated efficient electron transfer, reflected by increased current responses at higher scan rates and low charge transfer resistance values. Electrochemical characterization via cyclic voltammetry and electrochemical impedance spectroscopy (EIS) revealed that rice straw-powered cell exhibited the lowest charge transfer resistance (3.37 Ω), correlating with the highest recorded electrical current (9.23 ± 0.25 mA). Conversely, the microbial electrolysis cell (MEC) results showed that rice straw hydrolysate was the optimal substrate for biohydrogen production, yielding a maximum rate of 9.26 ± 0.08 mmol/day.L and a COD removal of 94.92 ± 0.88%, significantly outperforming xylan-based systems and the control cells. These findings indicate that while simpler carbohydrate structures like xylan favor electricity generation in MFCs, the bioavailable nutrients in alkaline-pretreated hydrolysates are more effective for electrohydrogenesis in microbial electrolysis cells. This research provides a critical comparative framework for selecting biomass fractions and system configurations to maximize the efficiency of agricultural waste-to-energy conversion strategies.

## Introduction

Burning fossil fuels releases CO_2_, which is a major cause of global warming^1^. This situation encouraged scientists to focus on research, which may replace conventional fossil fuels with an alternative source of energy. Agriculture lignocellulosic biomass is one of the annually readily available resources that are sustainable, with an estimated 200 billion tons produced globally^2^. Lignocellulosic biomass is often wasted or burned, leading to environmental pollution. However, recent advancements in technology and techniques have enabled the conversion of this waste into various energy sources^3^.

Rice straw is so readily available worldwide and a renewable source of lignocellulosic biomass. Rice straw is a significant secondary agricultural lignocellulosic biomass. It is mainly composed of cellulose, hemicellulose, and lignin, with trace amounts of inorganic components^4^. Conventional methods for managing rice straw in agricultural fields include burning it, which has negative environmental and health impacts. Moreover, the ashes, which are thought to be beneficial for the soil, contain heavy metals and low amounts of nutrients, reducing soil fertility. Burning rice straw also contributes to air pollution by releasing particulate matter, volatile organic compounds, and harmful gases like NOx, SOx, CO, and CO_2_. This can cause health issues like asthma, allergies, cardiac arrest, stroke, respiratory issues, eye and throat irritations, and even cancer^5^.

Rice straw is made up of a heterogeneous complex of carbohydrate polymers. A pretreatment step is required to break the lignin barrier and decrease cellulose crystallinity. Alkaline hydrolysis is a process by which rice straw is broken into simple compounds. Although there are other methods, hydrolysis is a faster process. Rice straw could be subjected to a high-pressure, high-temperature steam blast during the hydrolysis process. To accelerate the breakdown process, the rice straw must be treated with an acidic or basic medium. The bulk of rice straw is reduced by 42% by weight and 75% by volume because of hydrolysis. Hemicellulose degrades into shorter polymers of pentoses because of its structure and weaker linkages, which make it more easily susceptible to hydrolysis^6^.

The alkali pretreatment was carried out by using hydroxides of sodium to pretreat rice straw biomass^7^. Delignification occurs during the alkali pretreatment process and is caused by hydroxyl ions. The alkali treatment of lignocelluloses can result in three different types of reactions: lignin condensations, lignin degradation and dissolution, and fragmentations. Side chains may be reduced in number or eliminated because of the infrequent connections in lignin. Alkali delignification produces small molecules like formaldehyde and methanol^8^.

Two kinds of bioelectrochemical systems, microbial fuel cells (MFCs) and microbial electrolysis cells (MECs), have been used to produce bioelectricity and biohydrogen and to remove waste from the environment^9^. Microbial fuel cells (MFCs) are one of the methods used to produce bioelectricity, and they have great potential due to their energy efficiency, mild operating conditions, and co-generation system that produces bioelectricity and bioremediation simultaneously^10^. The MFC uses an oxidation-reduction mechanism to directly transform the chemical energy stored within the biomass into electrical energy^11^. Electroactive microorganisms (EAMs) play a crucial role in the electron transfer mechanism by acting as metabolic agents and transferring electrons generated throughout the process to the anodic electrode. Through the use of an external circuit, these electrons are transmitted to the cathode, producing electricity^12^. Through MFCs, the substrate is directly transformed into electricity^12^. Anaerobic anode chamber and aerobic cathode chamber are separated by an anion exchange membrane (AEM), which is the fundamental component of microbial fuel cells. For the microbial electrolysis cell (MEC), a catalyzed hydrogen progression cathode, an anode, an anion exchange membrane, and an electrolyte are the essential components of MEC. The cathode chamber must be completely anaerobic for the chemical evolution of hydrogen to typically occur in order for MECs to operate properly^13^. A microbial anode and an “almost conventional” hydrogen evolution cathode make up the core of a MEC^14^. In the anode chamber, organic molecules are oxidized anaerobically with the aid of the electrohydrogenesis process. This study established a correlation among the predominant forms of rice straw: the intricate composition of lignocellulosic biomass represented by crushed rice straw; rice straw hydrolysate; the simpler xylan derived from rice straw, and the operational conditions in both microbial fuel cells and microbial electrolysis cells.

## Materials and methods

### Chemicals, bacterial culture and biomass

All chemicals used in this study were of analytical grade and were purchased from Sigma Aldrich, Egypt. Anaerobic sludge used in this study was provided by Alexandria East Sewage Treatment Plant, Alexandria, Egypt. The rice straw used in this study was gathered from a nearby farm owned by the family of Dr. Hanan Moustafa Abdallah Moustafa in the Behera Governorate, Egypt. Permission was granted to Mahmoud Bayoumi to enter the premises for the purpose of collecting and removing rice straw residuals. This permission was valid for one month, from September 15 to October 15, 2024. The collector agrees to utilize proper equipment and ensure the field is cleared of straw without causing damage to the soil or surrounding infrastructure. Using scissors, the rice straw was first cut into lengths of two to three centimeters. All pieces were dried in an oven set at 60 °C for three hours. The oven-dried pieces were eventually pulverized into fine particles (1–2 mm) in a blender and kept at room temperature in a plastic container for use as a substrate in the biochemical systems^15^.

### Chemical hydrolysis of rice straw to produce rice straw hydrolysate and xylan

According to previous studies, rice straw hydrolysate (RSH) was produced by alkaline hydrolysis of rice straw (RS). The crushed rice straw was swollen in a 4% (w/v) NaOH solution at 85 °C for three hours, maintaining a solid-to-liquid ratio of 1:10 (w/v), as part of the alkaline treatment. The resultant hydrolysate was neutralized with acetic acid (pH 7.0). The mixture was centrifuged at 5000 x g for 15 min. then filtered through cheesecloth to eliminate any insoluble matter^15^.

Xylan was extracted from rice straw hydrolysate via precipitation using three volumes of 95% ethanol followed by centrifugation at 5000 xg for 15 min according to previous work. The precipitate was suspended in four volumes of water, followed by freeze drying using an Alpha 1–2 lyophilizer.

### Microbial fuel cell construction and operation

Microbial fuel cells used in this study were constructed manually using neoprene rubber sheets along with acrylic sheets in the presence of graphite rods (8 cm x 1 cm) as both anode and cathode electrodes as described before^16–18^. The distance between the two electrodes was kept at 5 cm, and the two chambers were separated using a cation exchange membrane (CEM; NAFION 117). The two electrodes were connected externally using titanium wire (1 mm diameter), and external resistance varying from 10 KΩ to 10 Ω was placed between the two electrodes. The anolyte was prepared using synthetic media containing 1 g/L CH_3_COONa, 15 g/LNH_4_Cl, 50 g/L NaCl, 2 g/L CaCl_2_, 1.5 g/L MgSO_4_, 10 g/L NaHCO_3_, and 1 ml/L trace elements according to previous studies^16–18^. It’s worth mentioning that the cell powered by sodium acetate (CH_3_COONa) was named as control cell, while other cells containing lignocellulosic biomass fractions (as electron donor) namely, crushed rice straw, rice straw hydrolysate and xylan were tested separately. To adjust anolyte carbon to nitrogen ratio, urea beads were added from one time to another. Anolyte pH was adjusted to 8.5 using 0.1 M sodium hydroxide. Anaerobic sludge was used as biocatalysis for organic matter degradation, and it was used after activation and cultivation in the same synthetic medium used in anolyte preparation in presence of water hyacinth and multivitamins and it was kept at 37 °C in a controlled incubator (New Brunswick Scientific Co., Edison, N.J., USA). Water hyacinth is some kind of lignocellulosic biomass that enriched in cellulose and hemicellulose^19^, so it was used for sludge adaptation for further application in MFC/ MEC for rice straw and its fractions hydrolysis. The catholyte used was 100 mM of potassium dichromate dissolved in 250 mM of sulfuric acid, serving as an electron acceptor in place of oxygen. All MFC was kept in a controlled environment incubator shaker (New Brunswick Scientific Co., Edison, N.J., USA) at 100 rpm and 37 °C. All experiments were done in triplicate, and electrical voltage and current were monitored daily using a digital multimeter (model: UNI-T UT33C+). Chemical oxygen demand (COD) of all MFCs’ anolyte was measured using the Hacch system before and after treatment.

### Evaluation of microbial fuel cell performance

To evaluate the whole cell’s performance (MFC powered with RS, MFC powered with RSH, and MFC powered with xylan along with MFC powered with acetate as a control cell), both current and voltage were monitored daily using an Avometer. In addition, the chemical oxygen demand (COD) was monitored through the system’s operation using HACCH, where COD % removal was calculated based on initial and final readings. Experiments were carried out in triplicate, and the results were expressed as mean ± standard deviation.

The electrochemical characterization of the MFCs was performed after the system had successfully started up and reached a stable reaction condition (steady state). Polarization is the most common and effective method for evaluating MFC performance, but in this study, cyclic voltammetry (CV) with an Origa Flex Potentiostat (a modular and vertical multichannel device made in Know-How and originally manufactured in France) was used to describe electrochemical behavior. Cyclic voltammetry and electrochemical impedance spectroscopy measurements were performed on the MFC in a frequency range of 100 kHz to 0.01 mHz with an AC signal of 10 mV amplitude. Anode impedance spectra were obtained by employing the anode (graphite rod, 8 cm x 1 cm) as the working electrode and the cathode (graphite rod, 8 cm x 1 cm) as the counter electrode in the presence of Ag/AgCl (Hanna instrument) as a reference electrode in the anode chamber. Considering the scan rate of the experiment determines how quickly the applied potential is scanned, cyclic voltammetry was performed at various scanning rates (10, 25, 50, 75, and 100 mV/s) (based on the recommended scanning rate from zero to 100 mV/s)^20,21^. All measurements were performed at atmospheric pressure and room temperature, with a frequency of 0.01 Hz-100 MHz and a voltage range of 0 to 1000 mV. The data was collected using OrigaMaster 5 as published before^16–18^.

### Microbial electrolysis cell construction and operation

The microbial electrolysis cells utilized in this investigation are comparable to the previously published cell^18^ it was built using a cathode of carbon cloth supported on stainless steel mesh (5 cm X 10 cm) coated with 0.5 mg Pt/cm^2^ and an anode of graphite rod (8 cm x 1 cm). We employed a cation exchange membrane, like the one in MFC, to separate the anode and cathode compartments. Titanium wire (1 mm in diameter) was used as a current collector. Resistance was applied between the two electrodes and was reduced gradually from 10 KΩ to 10 Ω. The applied potential from the external power supply was 0.8 V. The same sludge used to inoculate anolyte of MFC was used to inoculate MEC. After the system was constructed successfully, anolyte and catholyte were prepared as previously reported^18^, except lignocellulosic biomass fractions namely; rice straw hydrolysate and xylan were used separately as an energy carrier. In this study, three MECs were constructed and operated in batch mode at room temperature under static conditions; one of them was powered with rice straw hydrolysate and the second one was energized with xylan. In addition to these two cells, MEC powered with acetate (1 g/L) was used as a control cell. Hydrogen gas generated during the experiments was collected via water displacement, and its concentration and production rate were measured as described before^13,22^ using MQ 8 sensor (http://www.learningaboutelectronics.com/Articles/MQ-8-hydrogen-sensor-circuit-with-arduino.php). All systems’ performance was evaluated through monitoring cell potential, electrical current, power density, COD removal, and the generated hydrogen production rate. Experiments were carried out in triplicate, and the results were expressed as mean standard deviation.

## Results and discussion

### Performance evaluation of microbial fuel cell

Three dual chambers microbial fuel cells powered with different lignocellulosic biomass fractions were used in this study: MFC powered with RS, MFC powered with RSH and MFC powered with xylan in addition to the control cell. In all cells, the used catholyte was 100 mM of potassium dichromate dissolved in 250 mM of sulfuric acid, serving as an electron acceptor in place of oxygen. This substitution offers a cost-effective and controllable alternative, as prior studies indicate that the dissolved oxygen concentration limited by its solubility in water and the energy required for external supply correlates directly with its function as an electron acceptor. Utilizing potassium dichromate can yield a higher theoretical redox potential and faster reduction rates compared to oxygen^16^.

**Fig. 1:**
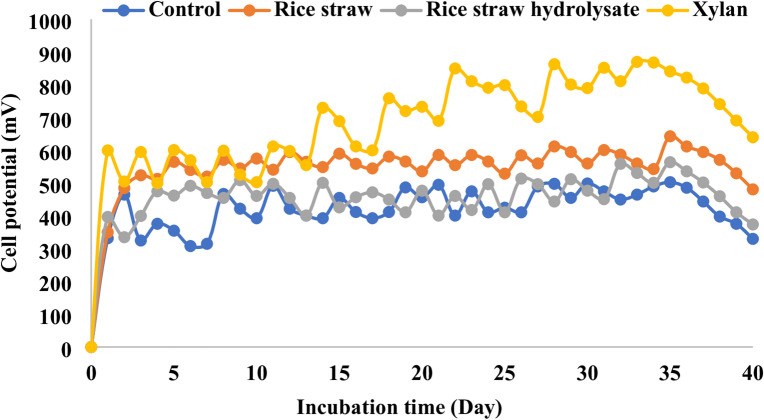
Closed circuit potential monitoring of microbial fuel cells.

**Fig. 2:**
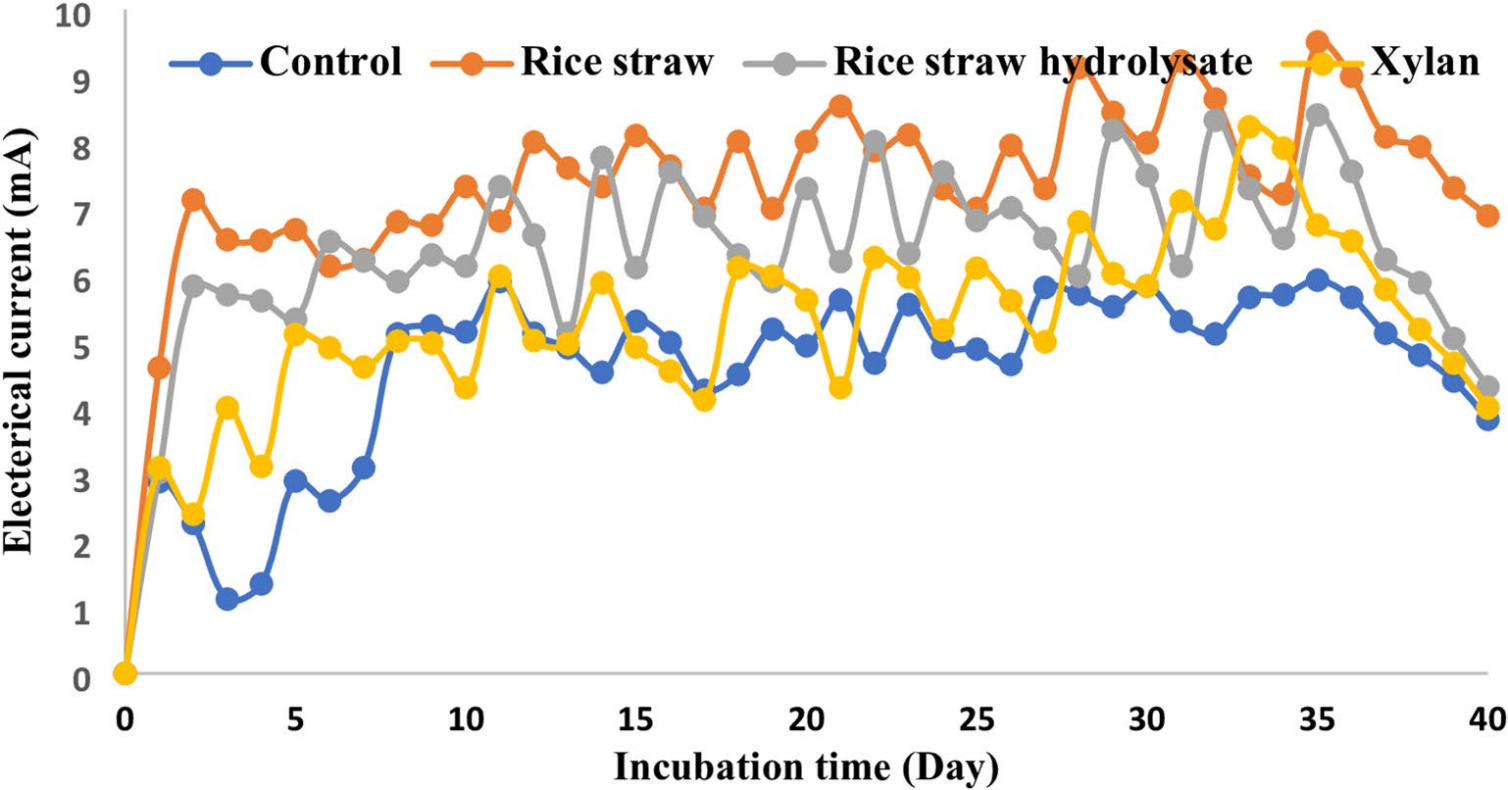
Electrical current monitoring of microbial fuel cells.

Figures 1 and 2 show cell potential and electrical current monitoring over time of the whole cells, respectively. The revealed results confirmed the ability of the bacterial consortium to consume all lignocellulosic biomass fractions under complete anaerobic conditions for electricity generation, where similar patterns of electricity generation were recorded that confirmed actual batch mode operation with fluctuation in electricity generation similar to the previous study^23^. Electricity generation using xylan is better than other fractions or even control cells (852 ± 27 mV cell potential compared to 630 ± 17, 532 ± 26, and 480 ± 25 mV in the case of rice straw, rice straw hydrolysate, and control cells, respectively) (Table 1). The power density generated using the whole cells is in the same line as cell potential, with the highest value of 8.88 ± 0.27 W/m^2^ using xylan as a substrate compared to 7.56 ± 0.18, 5.77 ± 0.21, and 3.53 ± 0.19 W/m^2^ in the case of rice straw, rice straw hydrolysate, and acetate, respectively (Table 1). Power density generated using xylan represent 2.41, 1.17, 1.5 fold over control cell, rice straw and rice straw hydrolysate respectively. It worth mentioning that the utilization of mixed bacterial culture (sludge), which has already acclimatized to the degradation of lignocellulosic biomass, play a significant role in enhancing power density through biomass utilization and its conversion to electrons, thus boosting energy generation, as indicated by prior studies^24–26^.

The reduced digestibility of rice straw and rice straw hydrolysate, in contrast to xylan, is attributable to the lignin content in rice straw and the presence of certain phenolic compounds in the hydrolysate that may impede microbial activity and, thus, digestion^27,28^.

The variation in biomass fraction digestibility is reflected on COD removal efficiency as it was; 97.88 ± 1.32% compared to 90.83 ± 1.02 and 87.23 ± 1.64 in case of rice straw and rice straw hydrolysate in respectively) (Table 1). Results are in the same line as previous studies about microbial inhibition due to presence of lignin and phenolic compounds in rice straw hydrolysate^29,30^ (Fig. 2).

**Table 1:** Summary of all microbial fuel cells performance regarding electricity generation and COD removal efficiency.

Parameter	Control cell	RS^1^ powered cell	RSH^2^ powered cell	Xylan powered cell
Cell potential (mV)	480 ± 25	630 ± 17	532 ± 26	852 ± 27
Electrical current (mA)	5.62 ± 0.29	9.23 ± 0.25	8.23 ± 0.24	8.12 ± 0.20
Power density (W/m^2^) ^*^	3.53 ± 0.19	7.56 ± 0.18	5.77 ± 0.21	8.88 ± 0.27
COD removal efficiency (%)	81.34 ± 2.13	87.23 ± 1.64	90.83 ± 1.02	97.88 ± 1.32

Results were expressed as mean $\:\pm\:$ standard deviation.

RS^1^: Rice straw.

RSH^2^: Rice straw hydrolysate.

Power density (W/m^2^) *: power density is power divided by electrode area.

One crucial electrochemical method for comprehending the principles underlying electron transfer in microbial fuel cells (MFCs) is cyclic voltammetry (CV). It aids in the identification of redox-active species, kinetics characterization, mechanism differentiation, electrode material evaluation, and monitoring microbial biofilm activity. Although CV is a diagnostic tool for optimizing MFC performance, its intricacy can make interpretation difficult. MFC processes can be better understood by combining CV with other analytical methods such as electrochemical impedance spectroscopy (EIS)^17,18,31^.

**Fig. 3:**
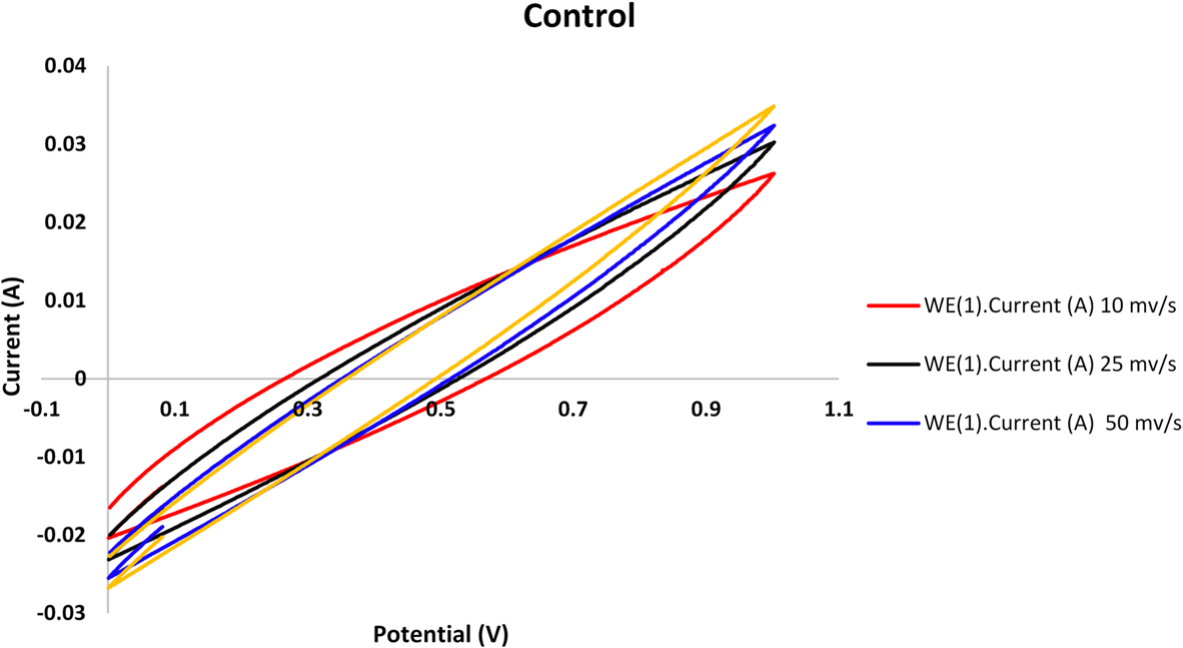
Control cell cyclic voltammograms obtained at different scan rates using a graphite rod anode.

**Fig. 4:**
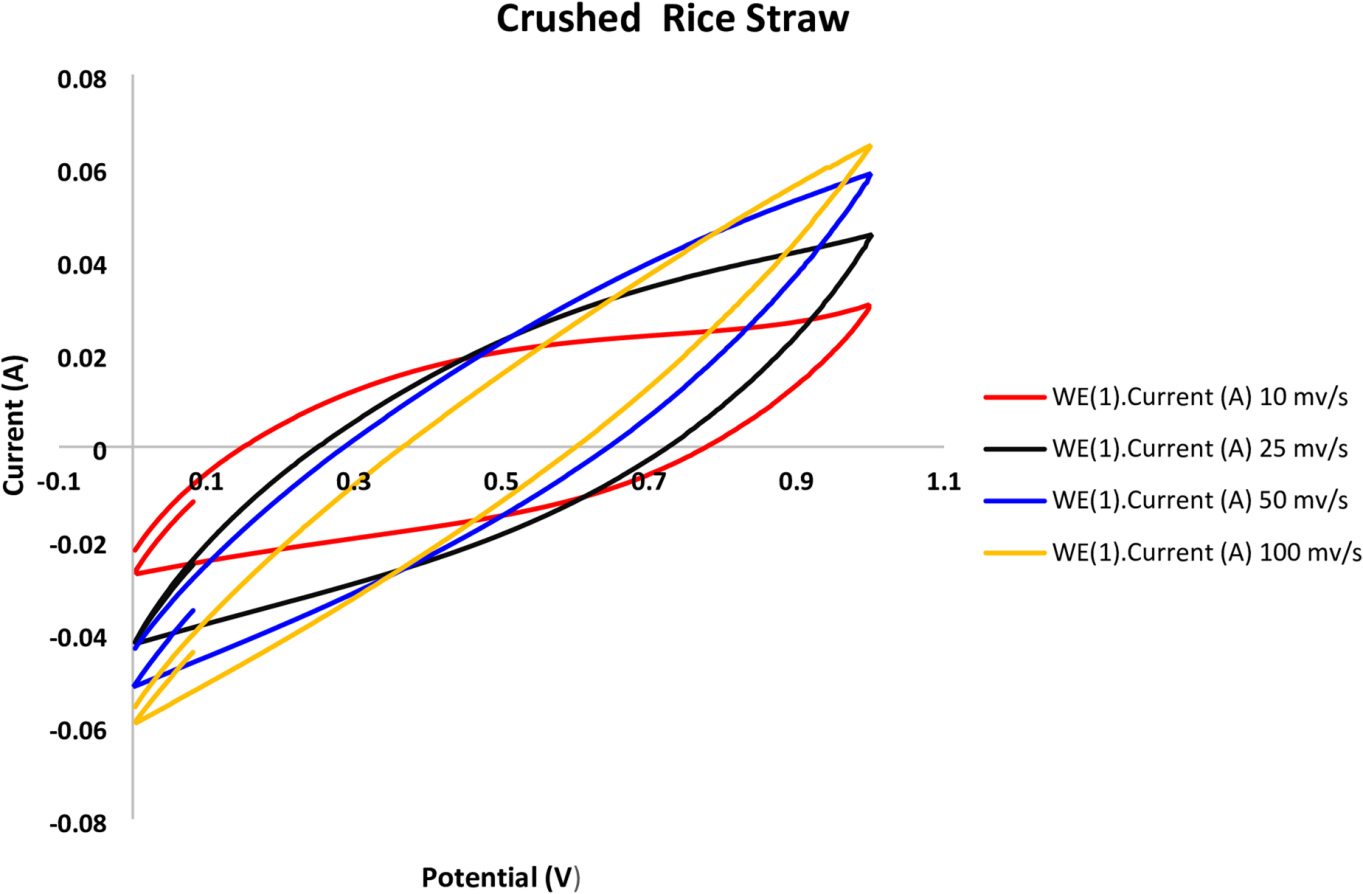
Crushed rice straw cell cyclic voltammograms obtained at different scan rates using a graphite rod anode.

**Fig. 5:**
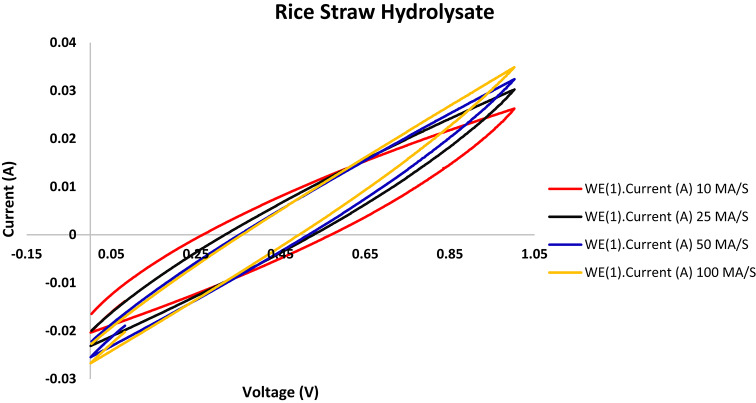
Rice straw hydrolysate cell cyclic voltammograms obtained at different scan rates using a graphite rod anode.

**Fig. 6:**
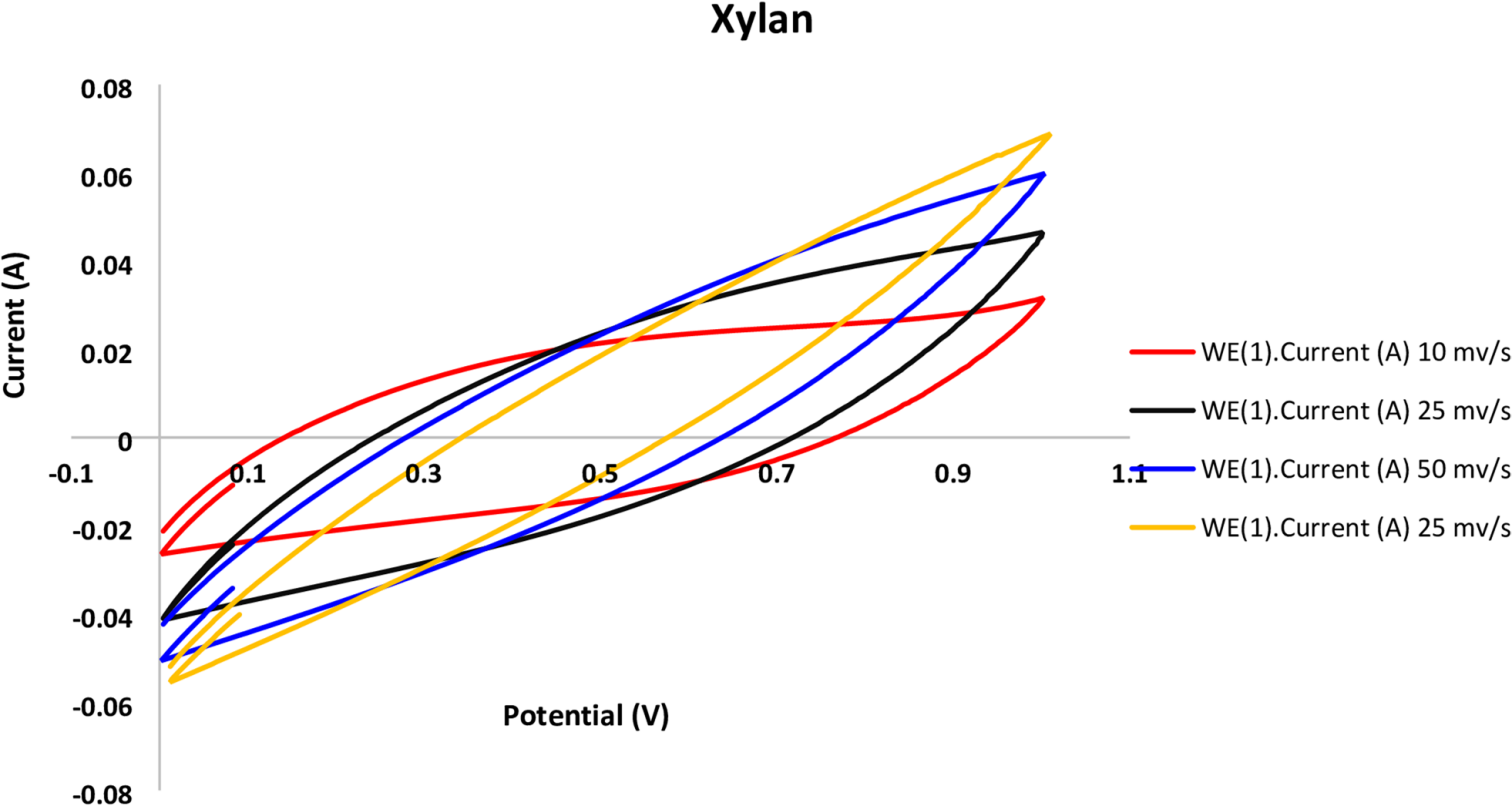
Xylan cell cyclic voltammograms obtained at different scan rates using a graphite rod anode.

Following the stabilization of the bioelectochemical systems, cyclic voltammetry was used to assess the performance of the MFCs utilized in this investigation at various scanning rates of 10, 25, 50, and 100 mV/s, as shown in Figs. 3, 4, 5 and 6.

The voltammograms reveal that quicker scanning rates and a higher potential window of 100 mV/s result in higher current compared to lower ones. Faster scan speeds boost electron transport, which raises currents, according to earlier research^32^. As previously noted^17^, there are many variations in MFC cyclic voltammograms, which can be attributed to various electrode materials, scanning parameters, and the conductivity of the anolyte and organic substrate.

**Fig. 7:**
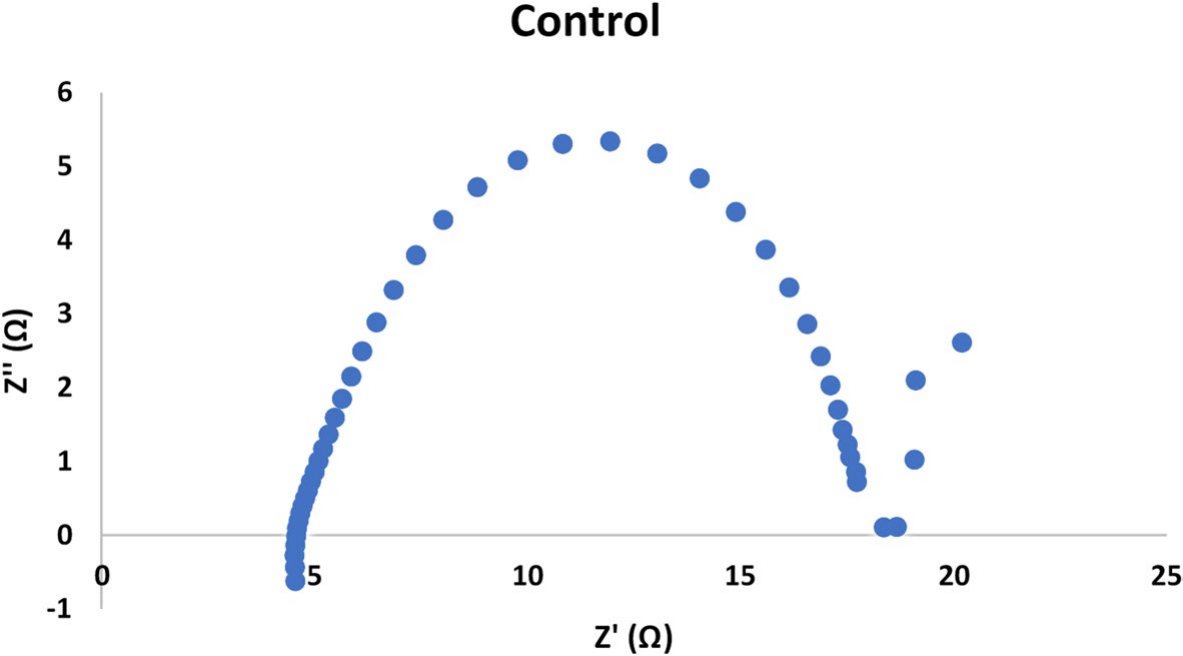
Electrochemical impedance spectroscopy of MFC-acetate (control).

**Fig. 8:**
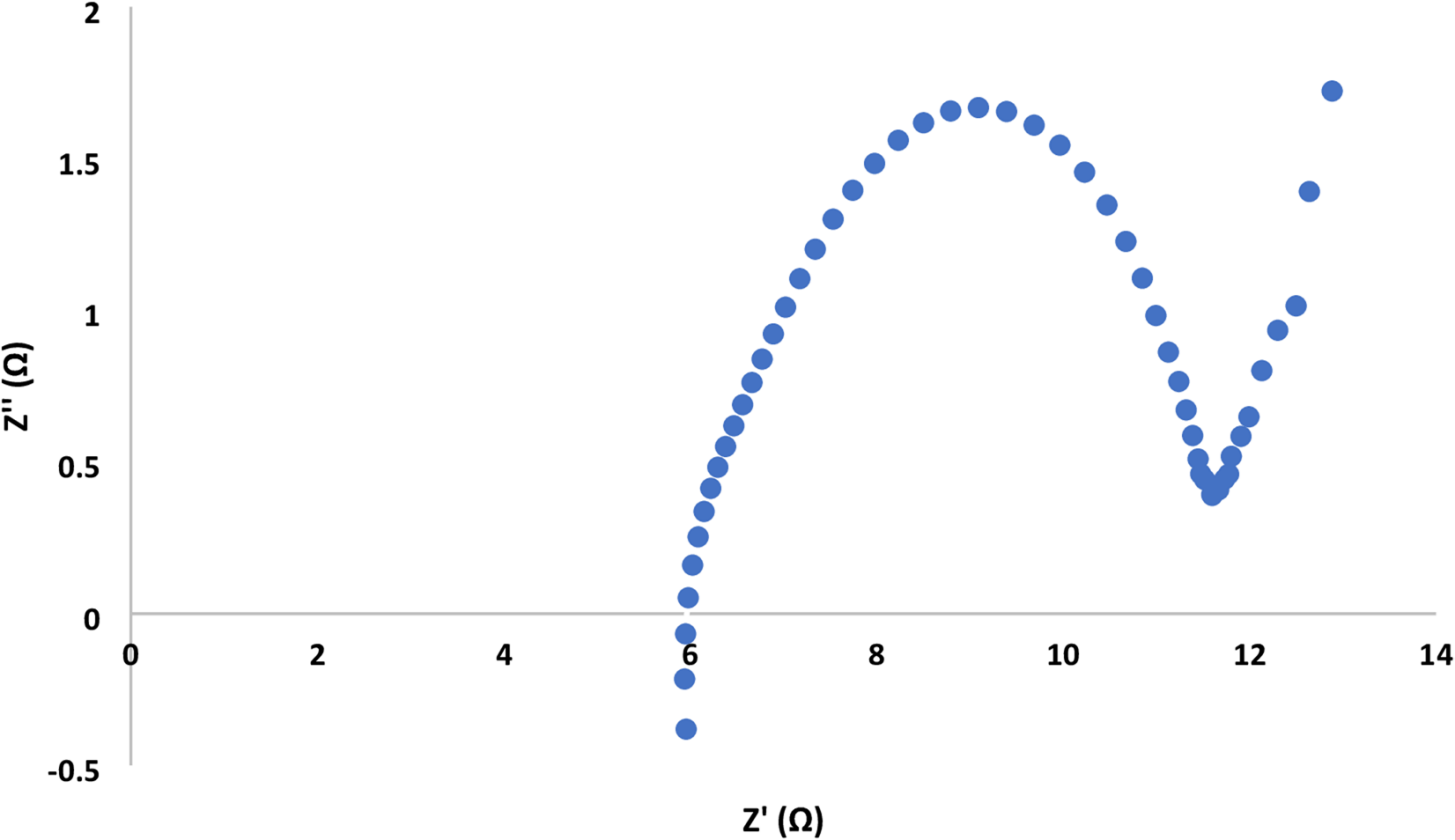
Electrochemical impedance spectroscopy of MFC-Rice straw hydrolysate.

**Fig. 9:**
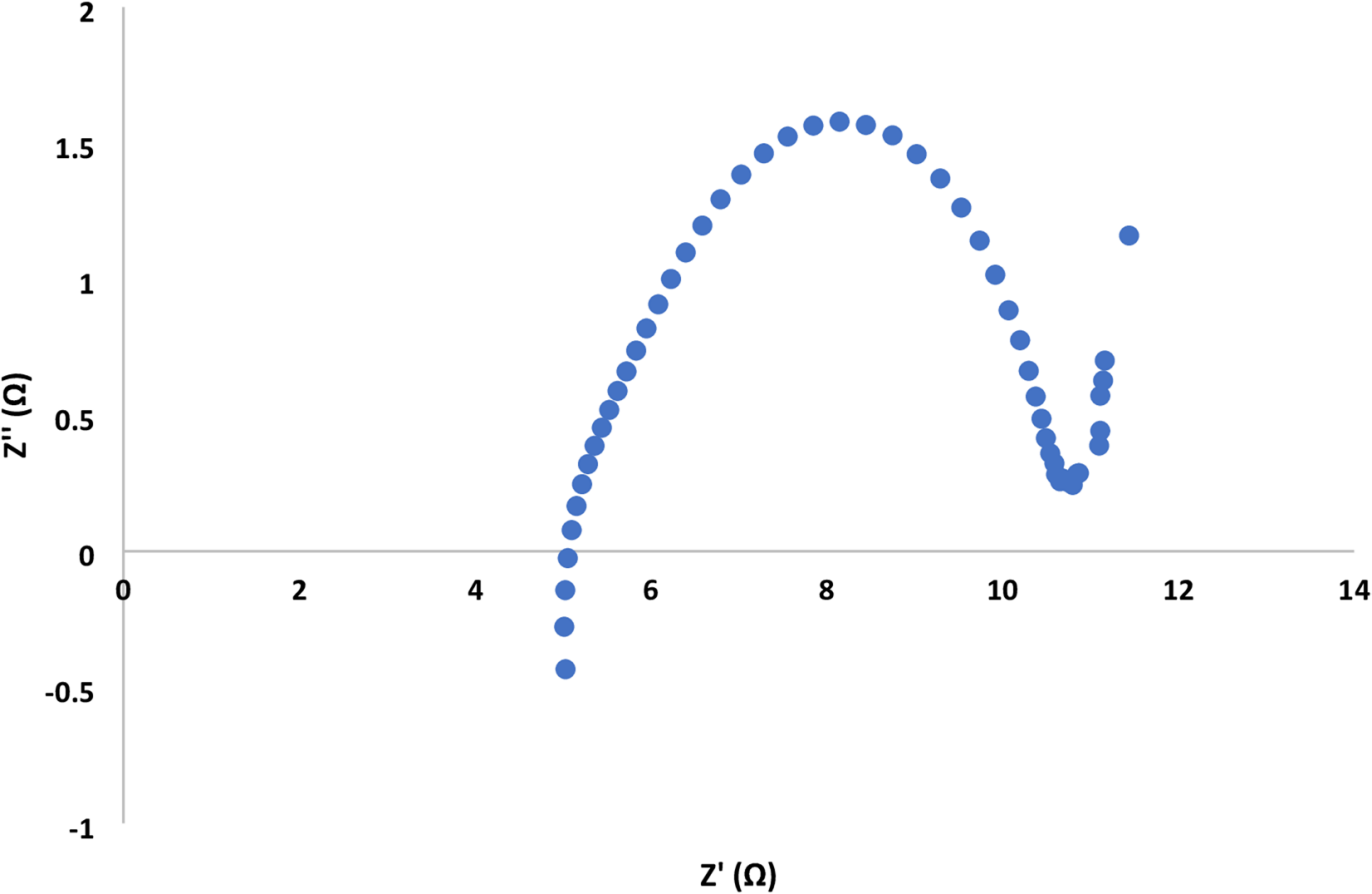
Electrochemical impedance spectroscopy of MFC- crushed rice straw.

**Fig. 10:**
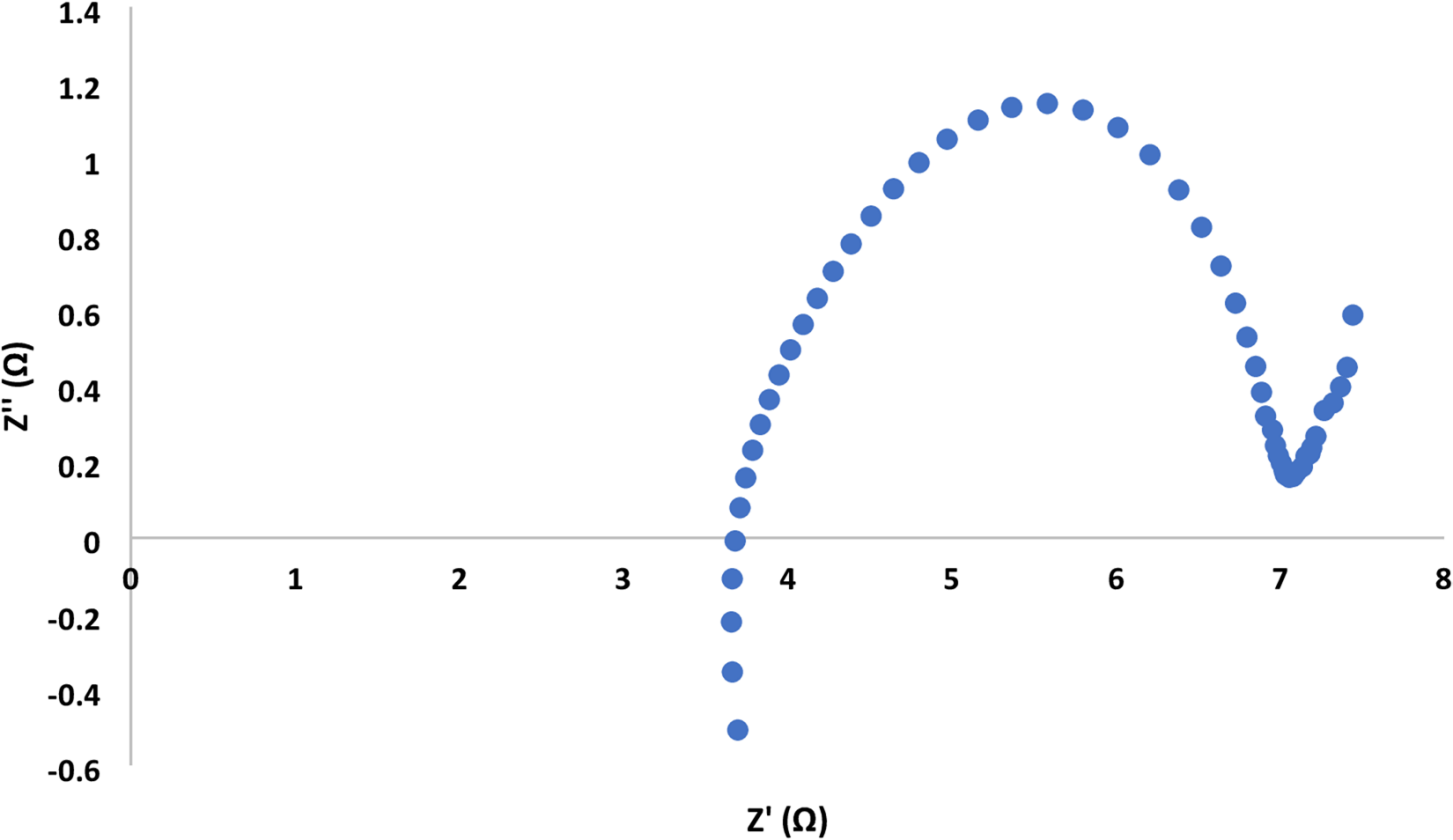
Electrochemical impedance spectroscopy of MFC- Xylan.

The Nyquist plot showed a semi circuit followed by linear relationship. Its diameter indicated the charge transfer resistance value. Figures 7, 8, 9 and 10 showed the charge transfer resistance were 14.1, 3.37, 5.64 and 5.05 Ω in the case of MFCs powered with acetate, RS, RSH and xylan in respectively. Results showed also that the lowest charge transfer resistance with MFC powered with RS. This finding was confirmed by the highest electrical current of 9.23 ± 0.25 mA. By contrast, acetate represented the highest charge transfer resistance which confirmed by the lowest electrical current of 5.62 ± 0.29 mA compared to other cells. It’s worth to mention that, the linear relation after semi circuit indicated easiest transfer of electrons from anolyte to electrode that confirmed the presence of non-mediated MFCs as previously explained^33^.

### Microbial electrolysis cell (MEC) performance for biohydrogen production from lignocellulosic biomass

**Fig. 11:**
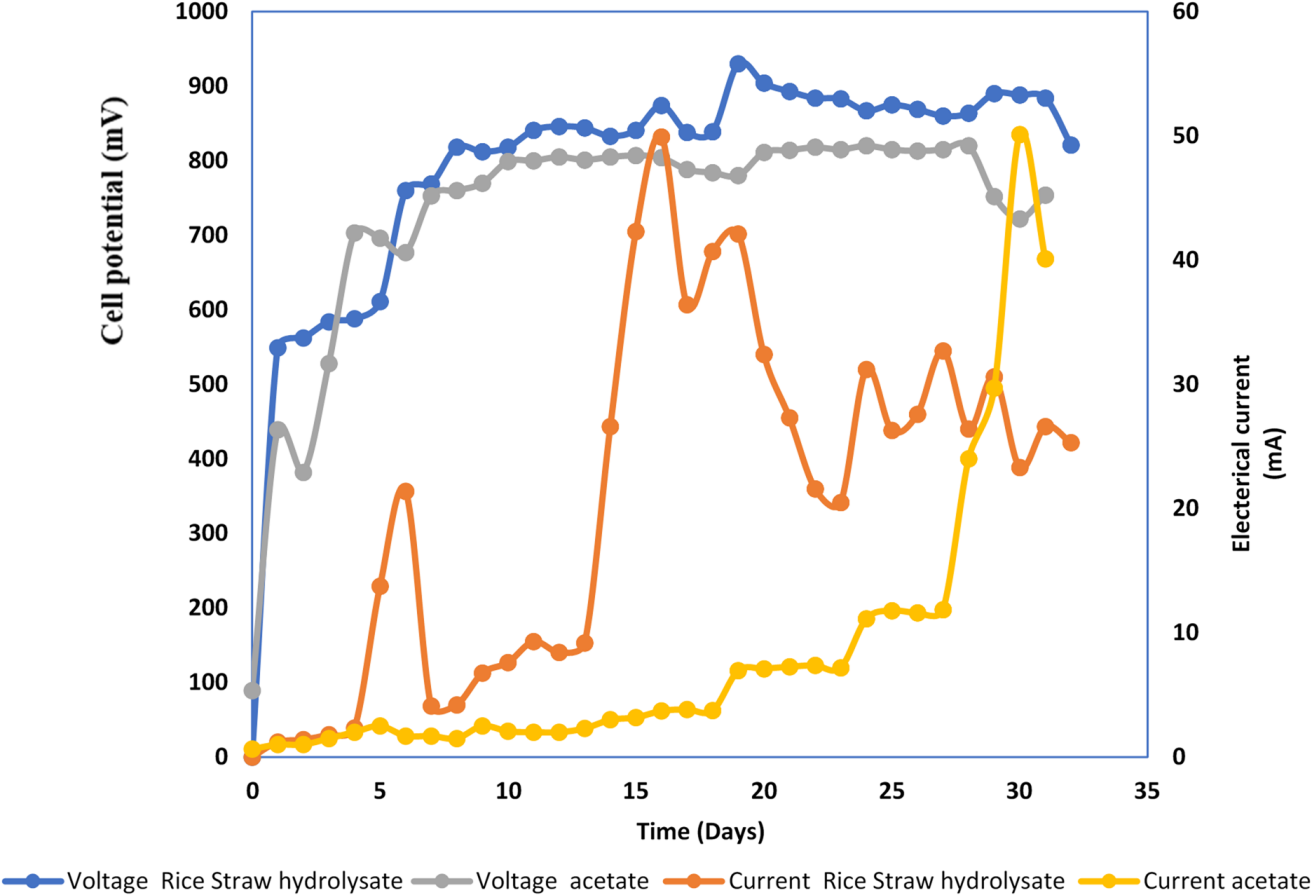
Voltage and current monitoring of control acetate-MEC versus rice straw hydrolysate-MEC.

**Fig. 12:**
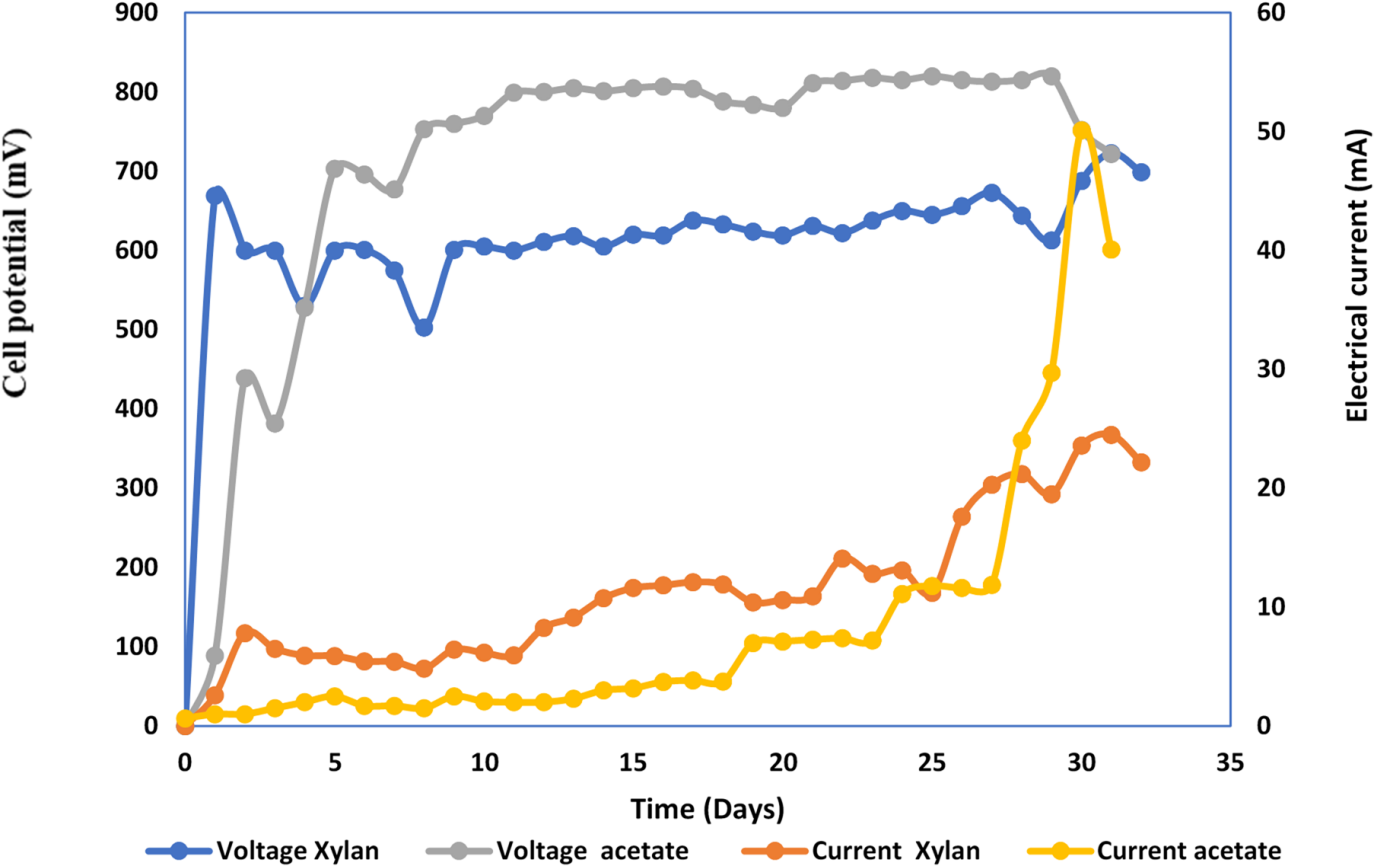
Voltage and current monitoring of control acetate-MEC versus Xylan-MEC.

**Fig. 13:**
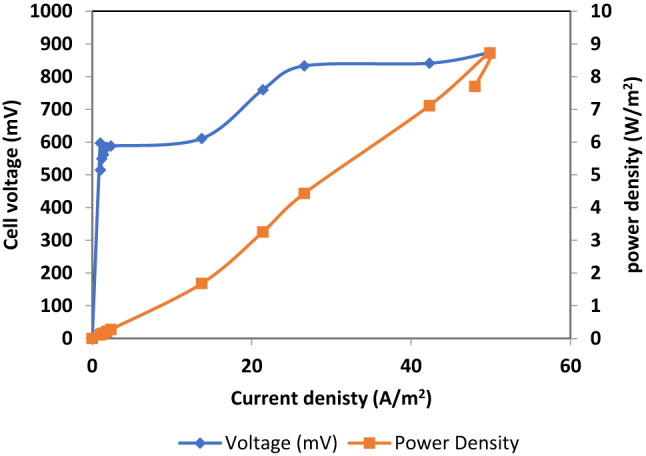
Voltage and current monitoring of control Rice straw hydrolysate-MEC versus Xylan-MEC.

**Fig. 14:**
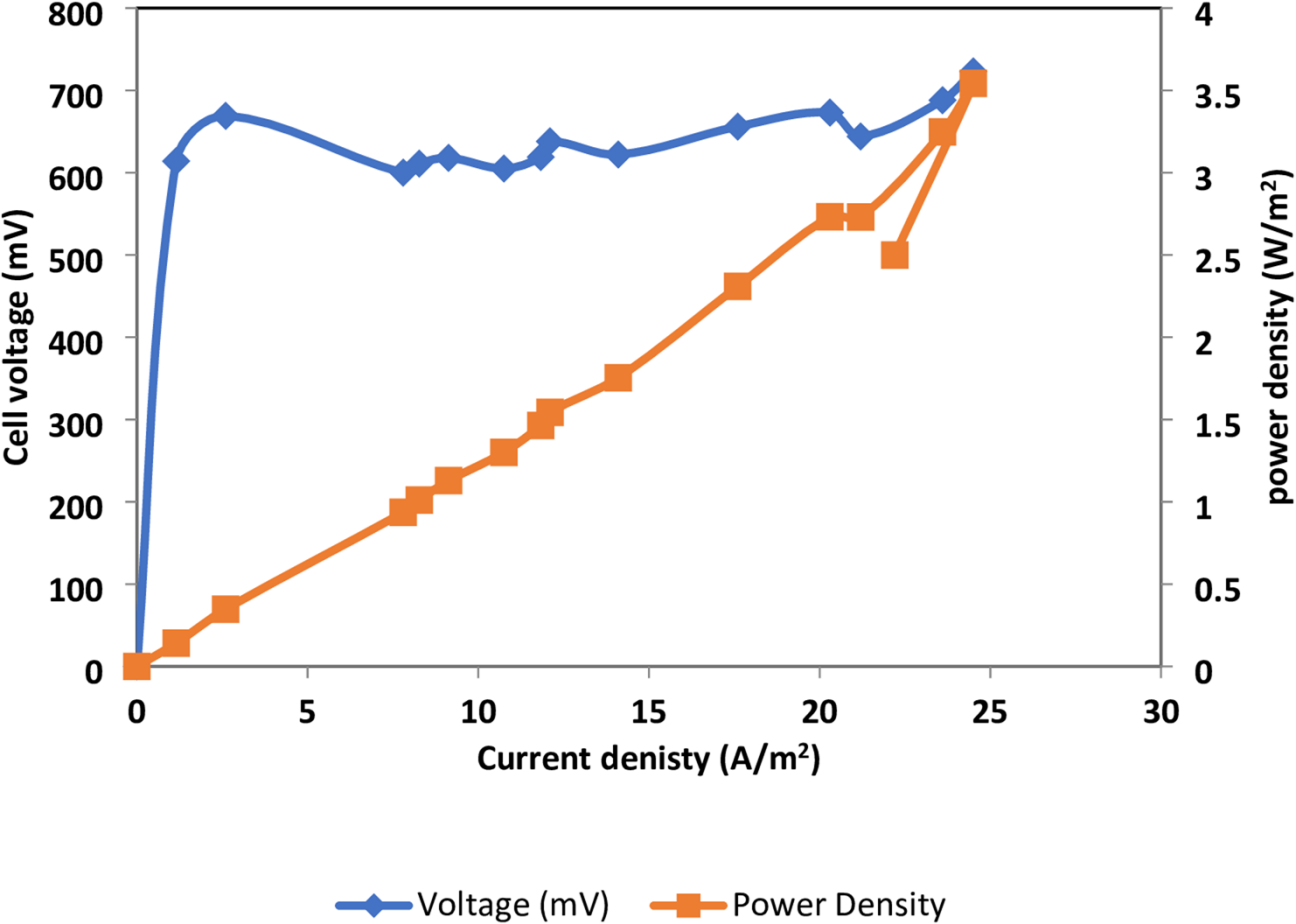
Determination of current and power density of MEC which is powered by rice straw hydrolysate.

Rice straw hydrolysate and xylan were utilized as substrates in the MEC, with sodium acetate serving as a control. Figure 11 demonstrates that rice straw hydrolysate MEC started with a big rise in cell potential, which eventually levels off between 709 ± 12 and 870 ± 7.5 mV. Conversely, the Xylan cell showed a beginning rise with subsequent stabilization of cell potential between 605 mV and 729 mV (Fig. 12). Figures (13-14) represent relation between power density (power per cathode area) versus cell potential and current density of both MEC- based hydrolysate and MEC- based xylan, respectively. Table 3 indicates power density (power per cathode area) of 8.22 ± 0.26 W/m^2^ for the hydrolysate and 3.52 ± 0.27 W/m^2^ for the xylan cell with the use of maximum current density (electric current per cathode area) and associated voltage. The conversion of xylan into a usable substrate by electroactive bacteria is a multi-step process requiring many different enzymes like endoxylanases and β-xylosidases^34^. This process often suffers from competition of methanogens, a type of microorganism, which can divert electrons and decrease current and power density in microbial electrolysis cells (MECs)^35^.

The anaerobic electrohydrogenesis process made hydrogen gas in direct relation to the amount of electricity it produced. The MQ-8 sensor measured the amount and purity of hydrogen gas after being calibrated with 99.999% pure hydrogen gas. The water displacement method was used to collect hydrogen that was generated during the 32-day MEC incubation period. Rice straw hydrolysate was used as a substrate. The largest quantity of hydrogen produced was 670 mL, and the highest rate was 9.26 ± 0.08 mmol/day. L with a COD removal efficiency of 94.92 ± 0.88%. Conversely, using xylan as a substrate produced 190 ml of hydrogen with an 82.27 ± 0.78% COD removal efficiency and a hydrogen production rate of 3.12 ± 0.13 mmol/day/L. It is important to note that using rice straw hydrolysate as a substrate reduces the rate of hydrogen production when using xylan by about one-third. The power density data, which showed a nearly one-third decrease when xylan was used instead of rice straw hydrolysate, are consistent with this conclusion. Since there is a direct correlation between the two, the decrease in power density causes the decrease in hydrogen production rate.

**Table 3:** Summary of MEC performance powered by several lignocellulosic biomass fractions (Results were expressed as mean $\:\pm\:$ standard deviation).

	Control	Rice Straw Hydrolysate	Xylan
Voltage (mV)	709 ± 12	870 ± 7.5	719 ± 9
Current (mA)	49.63 ± 0.23	48.99 ± 0.18	23.5 ± 0.37
Power density (W/m^2)^	7.12 ± 0.34	8.22 ± 0.26	3.52 ± 0.27
Hydrogen production rate (mmol/day)	3.56 ± 0.13	9.26 ± 0.08	3.12 ± 0.13
COD removal (%)	84 ± 1.2	94.92 ± 0.88	82.27 ± 0.78

## Conclusion

The potential of using lignocellulosic biomass derived from rice straw for sustainable bioenergy generation using microbial fuel cells (MFCs) and microbial electrolysis cells (MECs) was examined in this study. The results demonstrated that all tested lignocellulosic biomass fractions could be biologically converted under anaerobic conditions; however, system performance was strongly dependent on substrate composition and pretreatment. In MFCs, xylan showed superior electricity generation, achieving the highest cell potential, power density, and COD removal efficiency. This enhanced performance can be attributed to the simpler molecular structure of xylan, which facilitated microbial degradation and electron transfer. Electrochemical analyses using cyclic voltammetry and electrochemical impedance spectroscopy further confirmed effective electron transfer mechanisms and lower internal resistance in biomass-powered MFCs compared to the acetate control. On the other hand, MEC performance indicated that rice straw hydrolysate was the most efficient substrate for producing hydrogen. Alkaline pretreatment enhanced substrate bioavailability and encouraged electrohydrogenesis, as evidenced by the hydrolysate-based MEC’s highest power density, hydrogen production rate, and COD removal efficiency. The need for extra enzymatic hydrolysis steps and competition from methanogenic microbes were probably the causes of the lower hydrogen yields seen with xylan. This study highlights the importance of selecting appropriate biomass forms and bioelectrochemical system configurations to maximize energy recovery from lignocellulosic waste. The findings provide a comprehensive understanding of how substrate complexity influences bioelectricity and biohydrogen production pathways. This work contributes to the development of integrated waste-to-energy strategies and supports the sustainable management of agricultural residues such as rice straw.

## Data Availability

The datasets used and/or analyzed during the current study are available from the corresponding author upon reasonable request.

## References

[1] Shen, H., Wen, X. & Trutnevyte, E. Accuracy assessment of energy projections for China by energy information administration and international energy agency. *Energy Clim. Change*. **4**, 100111 (2023).

[2] Liang, J. et al. Promising biological conversion of lignocellulosic biomass to renewable energy with rumen microorganisms: a comprehensive review. *Renew. Sustain. Energy Rev.* **134**, 110335 (2020).

[3] Feng, Q. & Lin, Y. Integrated processes of anaerobic digestion and pyrolysis for higher bioenergy recovery from lignocellulosic biomass: a brief review. *Renew. Sustain. Energy Rev.* **77**, 1272–1287 (2017).

[4] Isikgor, F. H. & Becer, C. R. Lignocellulosic biomass: a sustainable platform for the production of bio-based chemicals and polymers. *Polym. Chem.* **6**, 4497–4559 (2015).

[5] Schikowski, T. et al. Ambient air pollution: a cause of COPD? *Eur. Respir. J.* **43**, 250–263. 10.1183/09031936.00100112 (2014). 23471349 10.1183/09031936.00100112

[6] Karimi, K., Shafiei, M. & Kumar, R. *Biofuel Technologies: Recent Developments* 53–96 (Springer, 2013).

[7] Veluchamy, C., Kalamdhad, A. S. & Gilroyed, B. H. Advanced pretreatment strategies for bioenergy production from biomass and biowaste. *Handbook Environ. Mater. Manage.* **2018**, 1–19 (2018).

[8] Zainab, A. K. et al. Beneficiation of renewable industrial wastes from paper and pulp processing. *Aims Energy*. **6**, 880–907 (2018).

[9] Unal, B. O., Arikan, E. B., Kumar, P. & Dizge, N. In *Bioelectrochemical Systems: Vol. 2 Current and Emerging Applications* 235–256 (Springer, 2021).

[10] Gajda, I., Greenman, J. & Ieropoulos, I. A. Recent advancements in real-world microbial fuel cell applications. *Curr. Opin. Electrochem.* **11**, 78–83 (2018). 31417973 10.1016/j.coelec.2018.09.006 PMC6686732

[11] Yu, Y. Y. et al. Single cell electron collectors for highly efficient wiring-up electronic abiotic/biotic interfaces. *Nat. Commun.* **11**, 4087 (2020). 32796822 10.1038/s41467-020-17897-9 PMC7429851

[12] Geng, B. Y. et al. Potential of Zymomonas mobilis as an electricity producer in ethanol production. *Biotechnol. Biofuels*. **13**, 36 (2020). 32158500 10.1186/s13068-020-01672-5 PMC7057670

[13] Varanasi, J. L., Veerubhotla, R., Pandit, S. & Das, D. Biohydrogen production using microbial electrolysis cell: recent advances and future prospects. *Microbial Electrochem. Technol.* **2019**, 843–869 (2019).

[14] Rousseau, R. et al. Microbial electrolysis cell (MEC): strengths, weaknesses and research needs from electrochemical engineering standpoint. *Appl. Energy*. **257**, 113938 (2020).

[15] Khalil, A. I., Hassan, M. M. & Moustafa, H. M. A. Valorization of rice straw xylooligomers for biohydrogen production via cell-free synthetic enzymatic pathway. *Biomass Convers. Biorefinery*. **15**, 19931–19945. 10.1007/s13399-025-06491-y (2025).

[16] Mohamed, A. A. R. et al. Performance evaluation of microbial fuel cell fabricated using green nano-graphene oxide as coating anode material. *Biomass Convers. Biorefinery* **2023**, 1–14 (2023).

[17] Mohamed, A. A. R. et al. Performance evaluation of microbial fuel cell fabricated using green nano-graphene oxide as coating anode material. *Biomass Convers. Biorefinery*. **15**, 297–310 (2025).

[18] El Salamony, D. H., Hassouna, M. S. E., Zaghloul, T. I., He, Z. & Abdallah, H. M. Bioenergy production from chicken feather waste by anaerobic digestion and bioelectrochemical systems. *Microb. Cell. Fact.* **23**, 102 (2024). 38575972 10.1186/s12934-024-02374-5 PMC10996200

[19] Sharma, A. & Aggarwal, N. K. *Water Hyacinth: A Potential Lignocellulosic Biomass for Bioethanol* (Springer, 2020).

[20] Bard, A. J. & Faulkner, L. R. Fundamentals and applications. *Electrochem. Methods* **2**, 580–632 (2001).

[21] Savéant, J. M. *Elements of Molecular and Biomolecular Electrochemistry: An Electrochemical Approach to Eectron Transfer Chemistry* (Wiley, 2006).

[22] Li, T., Li, R. & Zhou, Q. The application and progress of bioelectrochemical systems (BESs) in soil remediation: a review. *Green. Energy Environ.* **6**, 50–65 (2021).

[23] Liu, F. et al. Microbial electrochemical ammonia recovery from anaerobic digester centrate and subsequent application to fertilize Arabidopsis thaliana. *Water Res.* **220**, 118667. 10.1016/j.watres.2022.118667 (2022). 35667170 10.1016/j.watres.2022.118667

[24] Chukwuma, O. B., Rafatullah, M. & Tajarudin, H. A. A review on bacterial contribution to lignocellulose breakdown into useful bio-products. *Int. J. Environ. Res. Public Health*. **18**, 6001 (2021). 10.3390/ijerph18116001 PMC8199887 34204975

[25] Manyi-Loh, C. E. & Lues, R. Anaerobic digestion of lignocellulosic biomass: substrate characteristics (challenge) and innovation. *Fermentation* **9**, 755 (2023).

[26] Mohd Yusoff, M. Z. et al. Influence of pretreated activated sludge for electricity generation in microbial fuel cell application. *Bioresour. Technol.* **145**, 90–96. 10.1016/j.biortech.2013.03.003 (2013). 23566463 10.1016/j.biortech.2013.03.003

[27] Chen, X. et al. Tapping the bioactivity potential of residual stream from its pretreatments may be a green strategy for low-cost bioconversion of rice straw. *Appl. Biochem. Biotechnol.* **186**, 507–524 (2018). 29658061 10.1007/s12010-018-2751-1 PMC6209036

[28] Wang, X. et al. Inhibitory effects of phenolic compounds of rice straw formed by saccharification during ethanol fermentation by Pichia stipitis. *Bioresour. Technol.* **244**, 1059–1067 (2017). 28851161 10.1016/j.biortech.2017.08.096

[29] van der Maas, L., Driessen, J. L. & Mussatto, S. I. Effects of inhibitory compounds present in lignocellulosic biomass hydrolysates on the growth of Bacillus subtilis. *Energies* **14**, 8419 (2021).

[30] Fernández-Sandoval, M. et al. Removal of phenolic inhibitors from lignocellulose hydrolysates using laccases for the production of fuels and chemicals. *Biotechnol. Prog.* **40**, e3406 (2024). 37964692 10.1002/btpr.3406

[31] Gimkiewicz, C. & Harnisch, F. Waste water derived electroactive microbial biofilms: growth, maintenance, and basic characterization. *J. Visual. Exp. JoVE* **2013**, 50800. 10.3791/50800 (2013). 10.3791/50800 PMC4106282 24430581

[32] Bard, A. J. & Faulkner, L. R. Methods involving forced convection-hydrodynamic methods. *Electrochem. Methods: Fundam. Appl.* **2**, 331–367 (2001).

[33] Abbas, S. K. & Anwar, M. S. Electrochemical Impedance Spectroscopy (EIS) Gamry Interface 1010E Potentiostat/Galvanostat/ZRA (2025).

[34] Motta, F., Andrade, C. & Santana, M. A. Review of Xylanase Production by the Fermentation of Xylan: classification. In *Sustainable degradation of lignocellulosic biomass: Techniques, applications and commercialization. London, UK* 251–276 (IntechOpen, 2013).

[35] Yan, D., Yang, X. & Yuan, W. Electricity H2 generation from hemicellulose by sequential fermentation and microbial fuel/electrolysis cell. *J. Power Sources*. **289**, 26–33 (2015).

